# Indirubin alleviates CCl_4_-induced liver fibrosis by regulation of TGF-β-mediated signaling pathways

**DOI:** 10.22038/IJBMS.2023.70476.15319

**Published:** 2023

**Authors:** Xiaoying Li, Yuanzhi Yao, Lin Wei

**Affiliations:** 1 College of Biology and Food Engineering, Huaihua University. Key Laboratory of Research and Utilization of Ethnomedicinal Plant Resources of Hunan Province, Hunan, 418000, China; 2 College of Basic Medicine, Guizhou University of Traditional Chinese Medicine, Guiyang, 550025, China; 3 Daosheng Biology (Shenzhen) Co., Ltd, Shenzhen, 518107, China

**Keywords:** CCl_4_, Fibrosis, Indirubin, Liver, Mouse, TGF-β

## Abstract

**Objective(s):**

Liver fibrosis is a common liver disease caused by chronic liver damage. However, there are currently no approved drugs available to treat it. Therefore, the therapeutic effect of indirubin on liver fibrosis was evaluated. This study investigated the protective effect and related molecular mechanism of indirubin against CCl_4_-induced liver fibrosis in mice.

**Materials and Methods:**

We first detected the effect of indirubin on liver fibrosis in mice (n=8 per group, 32 mice total) by ELISA, HE, and Masson staining. Subsequently, the proliferation of activated HSCs was detected by MTT and EdU. Finally, the changes of related proteins and signaling pathways in mice treated with indirubin were investigated by qRT-PCR and Western blot. One-way ANOVA or two-tailed student’s t-test was used for comparison between groups.

**Results:**

Firstly, we found that indirubin (25 mg/kg) therapy could attenuate liver injury and significantly down-regulate α-SMA (*P*=0.0038) and collagen 1 (*P*=0.0057) in the liver using CCl_4_-induced liver fibrosis in mice. Secondly, we showed that indirubin (25 μM) could significantly inhibit hepatic stellate cell (HSC) trans-differentiation into myofibroblasts and proliferation (*P*=0.0063) in HSC-T6 cells treated by TGF-β. Finally, we showed that indirubin could greatly reduce the protein levels of p-Smad2/3, p38, p-ERK, and p-JNK *in vivo* and *in vitro.*

**Conclusion:**

Our results suggested that indirubin alleviated liver fibrosis and HSC activation mainly through TGF-β-mediated signaling pathways *in vivo* and *in vitro*. In conclusion, our data showed that indirubin could be a promising clinical therapeutic drug for the prevention and treatment of liver fibrosis.

## Introduction

Chronic liver injury leads to liver fibrosis, which is caused by various pathogenic processes, including viral infection, alcohol abuse, and nonalcoholic fatty liver disease (NAFLD) (1, 2). The fibrotic liver is characterized by excessive accumulation of extracellular matrix (ECM) poteins including collagen 1 (3-5). 

In the liver, activated Kupffer cells secret transforming growth factor-β (TGF-β), which causes hepatic stellate cell (HSCs) activation (4). Then, HSCs transdifferentiate into collagen-secreting myofibroblasts and promote liver fibrosis progression (6, 7). Therefore, TGF-β is considered a major fibrotic growth factor in accelerating the progression of liver fibrosis (3, 8-10). TGF-β stimulates HSCs activation by activating Smads pathways (Smad2/3), mitogen-activated protein kinases (MAPKs), as well as NF-kB signaling pathways in HSCs (11). Recent studies have demonstrated that the anti-fibrotic effects could be achieved through inhibition of TGF-β signaling pathways (5, 12-15). However, there are still no effective drugs to treat liver fibrosis. Thus, it is urgent to develop a suitable pharmacology to suppress HSCs activation for the treatment of liver fibrosis.

Indirubin, a bis-indole alkaloid, is usually found in Indigo plants or mollusks of the family *Muricidae* (16, 17). Several investigations about indirubin mainly focused on tumor diseases, which indicated that it could arrest the cell cycle, inhibit cell proliferation and induce apoptosis in ovarian cancer and chronic myelogenous leukemia (CML)(18-20). In addition, indirubin also can be used to alleviate psoriasis and idiopathic pulmonary fibrosis (IPF)(16). However, it remains unclear whether indirubin is involved in HSCs activation and liver fibrosis progression. Therefore, it is very necessary to study whether indirubin has the effect of treating liver fibrosis.

In this study, we used carbon tetrachloride (CCl_4_) to induce liver fibrosis in mice and TGF-β to activate HSC-T6 cells, and then evaluated the effects of indirubin on *in vivo* mouse model and *in vitro* HSC-T6 cells. Our results show that indirubin has a protective effect on liver fibrosis and HSC-T6 cell activation. Next, we show that indirubin plays a protective role in liver fibrosis by inhibiting TGF-β/Smads signaling. Together, our results suggest that indirubin may be a potential drug for the therapy of liver fibrosis in the clinical setting.

## Materials and Methods

***Animals and experimental design***

Male BALB/c mice (8 weeks old) were purchased from Beijing HFK Bioscience Co., LTD. Mice were housed under a 12 hr light–dark cycle with free access to water and food. All animal procedures were performed in accordance with the Guide for the Care and Use of Laboratory Animals and approved by the Animal Experimental Ethics Committee of Huaihua University (ethical code: HHTC-2021031258).

Mice were randomly distributed into four groups (n=8 per group) as follows: 1) control group, 2) CCl_4 _(Sigma-Aldrich, USA)-induced group, 3) 12.5 mg/kg indirubin (MedChemExpress, China) group (CCl_4_ + 12.5 mg/kg indirubin), and 4) 25 mg/kg indirubin group (CCl_4_ + 25 mg/kg indirubin). The CCl_4_-induced group was treated with CCl_4_ (20% CCl_4_/olive oil (Merck, Germany); 5 ml/kg) thrice a week for 8 weeks to induce liver fibrosis. In the indirubin-treated group, mice were administered CCl_4_ for 4 weeks and then administered indirubin at a dose of 12.5 or 25 mg/kg daily, together with CCl_4_ gavage for another 4 weeks. An equal volume of olive oil instead of CCl_4_ was given to rats by gavage as control (16).

***Alanine aminotransferase (ALT) and aspartate transaminase (AST) assessment***

For measuring serum levels of ALT and AST we utilized enzyme-linked immunosorbent assay (ELISA) commercial kits purchased from Ruixin Biotech (Quanzhou, China). The experimental steps were strictly performed according to the manufacturer’s manual (3).

***Histological assays***

The liver tissue was fixed in 4% paraformaldehyde (Merck, Germany) for 24 hr and was embedded with paraffin (Merck, Germany) after gradient-alcohol dehydration, xylene vitrification, and waxdip. Liver sections were used for hematoxylin/eosin (HE) staining and Masson’s trichrome (Solarbio Life Sciences, China) staining study (3).

***Cell culture and treatment***

HSC-T6 cells were purchased from Pricella (Wuhan, China). The cells were maintained in Dulbecco’s modified Eagle’s medium (DMEM; Thermo Fisher Scientific; USA) supplemented with 10% fetal bovine serum (FBS; Hyclone; USA) and 1% penicillin-streptomycin (HyClone, USA) at 37 °C with 5% CO_2_.

To evaluate the effect of indirubin on cell viability and proliferation, HSC-T6 cells were seeded in a 96-well plate with 1×10^4^ cells/ml per well and incubated for 24 hr before the addition of TGF-β. Cells were incubated for 24, 48, and 72 hr and treated with different concentrations of indirubin (5, 10, and 25 μM). The effects of indirubin on HSC viability and proliferation were tested by 3-(4,5-dimethyl-thiazol-2-yl)-2,5-diphenyl-tetrazolium bromide (MTT, Sigma-Aldrich, USA) assay and 5-ethynyl-2’-deoxyuridine (EdU) (Ribobio, Guangzhou, China) assay, respectively (21). The MTT and EdU assays were performed according to the manufacturer’s instructions.

To evaluate the effect of indirubin on HSCs activation, cells were serum-starved overnight and then treated with TGF-β (5 ng/ml) in the presence or absence of indirubin (5, 10, and 25 μM) for 24 hr (6).

***Quantitative real-time PCR (qRT-PCR) analysis***

Total RNA was isolated from liver tissues and HSC-T6 cells using Trizol (TRizol reagent, Thermo Fisher, USA). 2 µg RNA was used to synthesize cDNA using the iScript™ cDNA Synthesis Kit (Bio-Rad, USA). qRT-PCR was done simultaneously using a qScript™ One-Step qRT-PCR Kit (Quanta Biosciences, USA). The samples were run on a LightCycler® 96 Instrument Real Time-PCR System (Roche Applied Science, Switzerland). The GAPDH gene was used as an internal control to normalize gene expression (4).

***Western blot (WB) analysis***

The samples were harvested and whole-cell lysates were prepared. The protein concentrations were measured using a bicinchoninic acid (BCA) protein assay kit (Beyotime, China). Proteins were identified by 12% SDS-PAGE and transferred to polyvinylidene fluoride (PVDF) (Merck, Germany) membrane. The membrane was blocked with 5% BSA in TPBS (0.05% Tween-20 in TBS solution) at room temperature for 1–2 hr. Subsequently, WB analysis was performed. Primary antibodies of anti-α-SMA (Proteintech, 14395-1-AP**, **China), anti-Collagen-1 (Proteintech, 14695-1-AP**, **China), anti-Smad2/3 (Abcam, ab202445**, **UK), anti-p-Smad2/3 (Abcam, ab254407), anti-ERK (Abcam, ab184699**, **UK), anti-p-ERK (Abcam, ab201015**, **UK), anti-p38 (Proteintech, 66234-1-Ig**, **China), anti-p-p38 (Proteintech, 28796-1-AP**, **China), anti-JNK (Proteintech, 66210-1-Ig**, **China), anti-p-JNK (Proteintech, 80024-1-RR**, **China) and anti-GAPDH (Proteintech, 10494-1-AP**, **China). The secondary antibody was added and then incubated at room temperature for 1.5 hr. Protein bands were performed with ECL Prime reagent and chemiluminescence signals were detected by Odyssey XF (LI-COR Biosciences)(6).

***Statistical analysis***

Data were presented as means±SD. Statistical analysis was performed using GraphPad Prism software version 6.04. One-way ANOVA or two-tailed student’s t-test was used for comparison between groups. Values of *P*<0.05 were considered statistically significant.

## Results

***Indirubin protects against CCl***_4_ ***-induced liver injury and fibrosis***

**Figure 1:**
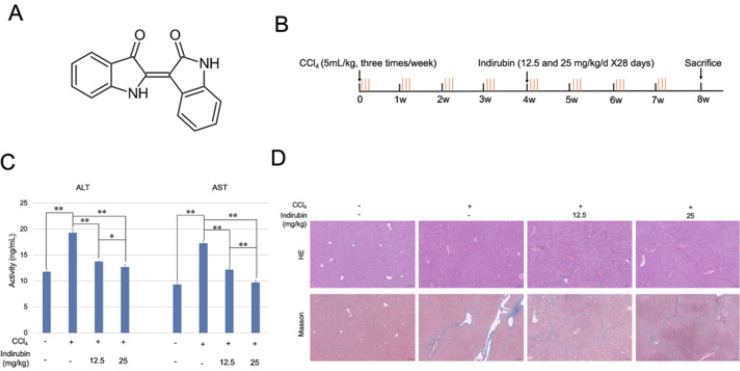
Indirubin protects against carbon tetrachloride (CCl_4_)-induced liver injury

The molecular structure of indirubin is shown in Figure 1A. To evaluate the therapeutic effects of indirubin on CCl_4_-induced liver fibrosis, the mice were treated with indirubin as illustrated in Figure 1B. Compared with the control group, the serum activities of ALT and AST in the CCl_4_-induced group significantly increased, and CCl_4_-induced mice with indirubin treatment (12.5mg/kg and 25mg/kg) had significantly reduced serum ALT and AST activities compared with CCl_4_-induced group (*P*<0.01) (Figure 1C). 

**Figure 2:**
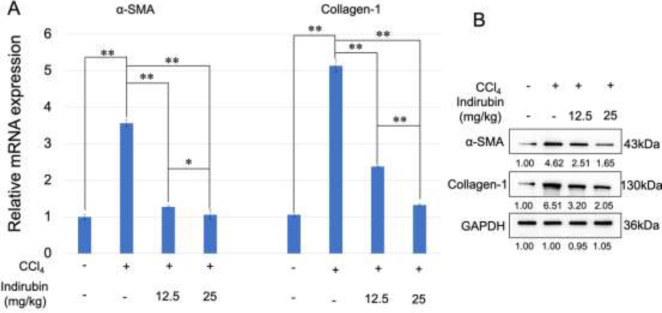
Indirubin alleviates carbon tetrachloride (CCl_4_)-induced liver fibrosis

Next, histological evaluation by HE staining and Masson’s trichrome staining showed severe liver fibrosis and collagen deposition in the CCl_4_-induced group, as compared with the control group. Interestingly, indirubin treatment markedly reduced collagen accumulation in the liver (Figure 1D). Furthermore, the mRNA expression of α-SMA and Collagen-1 was markedly increased in CCl_4_-induced mice but was decreased gradually in indirubin-treated mice (*P*<0.01) (Figure 2A). Additionally, the same trend was also observed at the protein level for α-SMA and Collagen-1 (Figure 2B). All these results suggested that indirubin could alleviate CCl_4_-induced liver fibrosis *in vivo*.

***Indirubin affects HSC-T6 cell proliferation in a dose-dependent manner***

**Figure 3:**
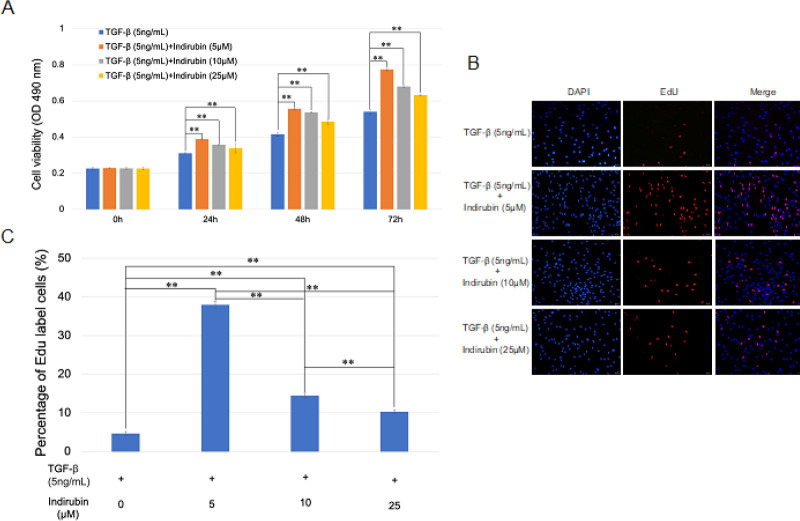
Indirubin affects HSC-T6 cell viability and proliferation in a dose-dependent manner

To examine how indirubin protects against liver fibrosis, we first investigated the effect of indirubin on cell 

viability and proliferation of HSC-T6 cells. HSC-T6 cells were treated with various concentrations (5 μM, 10 μM, and 25 μM) of indirubin for 0, 24, 48, and 72 hr after TGF-β treatment for 24 hr, and cell viability and proliferation were measured by MTT and EdU, respectively. The MTT assay showed that indirubin significantly inhibited cell viability in a dose-dependent manner. However, the inhibitory effect was not strongly related to the total dosing time (*P*<0.01) (Figure 3A). The EdU-labeling assay also indicated that indirubin inhibited cell proliferation in a dose-dependent manner in HSC-T6 cells (*P*<0.01) (Figures 3B and C). The results showed that indirubin could significantly inhibit the proliferation of HSC-T6 cells with higher doses showing stronger inhibition.

***Indirubin inhibits the TGF-β-induced HSC activation***

**Figure 4:**
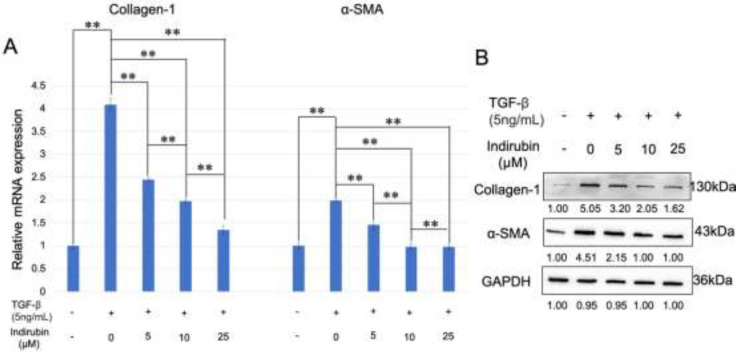
Indirubin inhibits the TGF-β-induced HSC activation

HSCs activation secretes a large amount of ECM proteins, which are deposited in the interstitial space of the liver, eventually leading to liver fibrosis. Therefore, the fundamental approach to treat liver fibrosis is to inhibit HSCs activation. The results showed that expression levels of Collagen 1 and α-SMA were significantly up-regulated after TGF-β treatment compared to the control group (*P*<0.01) (Figure 4A and B). However, the levels of Collagen 1 and α-SMA were significantly decreased after indirubin treatment compared to the TGF-β treatment group, suggesting that indirubin could inhibit TGF-β-induced HSCs activation (*P*<0.01) (Figure 4A and B). The above results indicated that indirubin could also inhibit the activation of HSCs *in vitro*, which is consistent with our *in vivo* results.

***Indirubin inhibits TGF-*** ***β*** ***-mediated signaling pathways***

**Figure 5:**
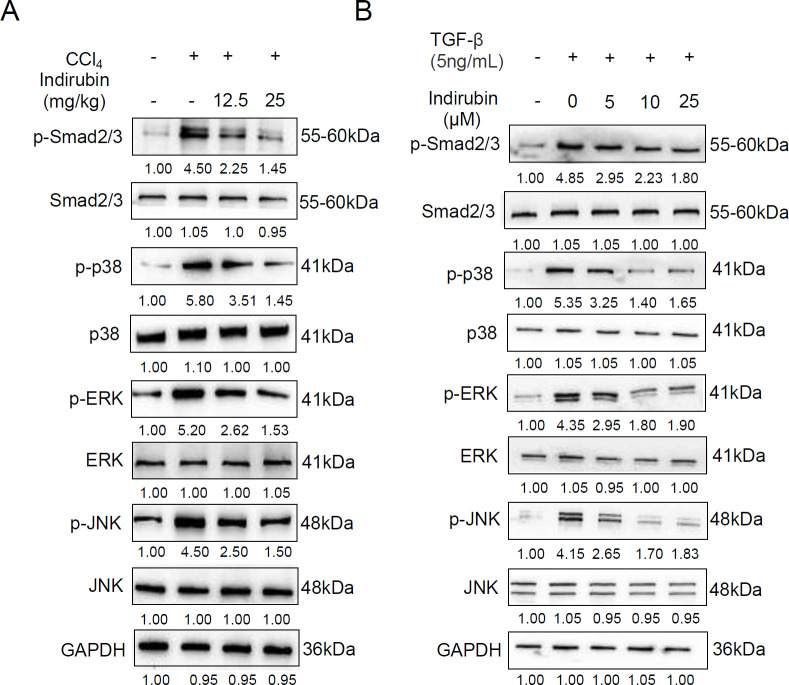
Indirubin inhibits TGF-β-mediated signaling pathways *in vivo* and *in vitro*

Previous studies suggested that TGF-β-mediated signaling pathways play an important role in regulating the differentiation of fibroblast to myofibroblast, we next investigated the effect of indirubin on TGF-β-induced pro-fibrogenic signaling in CCl_4_-induced liver fibrosis. We first examined the effect of indirubin on TGF-β/Smads signaling in CCl_4_-induced liver fibrosis. The p-Smad2/3 was investigated to explore whether indirubin could alleviate the TGF-β/Smad signaling pathway in mice after CCl_4_-induced liver fibrosis. The results showed that the expression of p-Smad2/3 was significantly up-regulated after CCl_4_-induced liver fibrosis and was attenuated by indirubin treatment in a dose-dependent manner (Figure 5A). In addition, the MAPK signaling pathways were also implicated in fibroblast differentiation induced by TGF-β. We then investigated whether indirubin could suppress TGF-β-mediated MAPK signaling. The results showed that indirubin attenuated CCl_4_-induced MAPKs (p38, p-ERK, p-JNK) activity (Figure 5A). These results indicated that indirubin significantly inhibited TGF-β signaling via the suppression of Smad and MAPK signaling factors *in vivo*.

To further explore the effect of indirubin on HSCs activation, we also detected the TGF-β-mediated signaling pathways in HSC-T6 cells. As expected, TGF-β induced a significant increase of p-Smad2/3 in HSC-T6 cells and indirubin could down-regulate the expression of p-Smad2/3 induced by TGF-β in a dose-dependent manner (Figure 5B). Furthermore, indirubin also attenuated TGF-β-induced MAPKs (p38, p-ERK, p-JNK) activity (Figure 5B). These results indicated that indirubin markedly blocked TGF-β signaling pathways via inhibition of Smad and MAPK signaling factors* in vitro*. Taken together, the above results suggested that indirubin might target the TGF-β-mediated signaling pathways to prevent liver fibrosis *in vivo *and *in vitro*.

(A) Chemical structure of indirubin. (B) Experimental design scheme. (C) Serum ALT and AST activities. (D) HE staining and Masson's trichrome staining of liver sections (Scale bar: 100 μm). The data are presented as mean±SD. *P*-values: * *P*<0.05, **P<0.01 using the two-tailed Student’s t-test

ALT: Alanine aminotransferase; AST: Aspartate transaminase

(A) mRNA expression levels of α-SMA and Collagen 1 were significantly decreased after indirubin (12.5 mg/kg, 25 mg/kg) treatment. The data are presented as mean±SD. *P*-values: * *P*<0.05, **P<0.01 using the two-tailed Student’s t-test

(B) Protein expression levels of α-SMA and Collagen 1 were significantly decreased after indirubin (12.5 mg/kg, 25 mg/kg) treatment

(A) Effects of indirubin (5 μM, 10 μM and 25 μM) on HSC-T6 cells viability. Cell viability was assessed by MTT assay. (B) Effects of indirubin (5 μM, 10 μM and 25 μM) HSC-T6 on cells proliferation. Cell proliferation was assessed by EdU assay. DAPI (bule), EdU incorporation (28). Scale bar: 50 μm). (C) Quantification of EdU incorporation. Data presented as percent cells with EdU staining and include three biological replicates. The data are presented as mean ± SD. *P*- values: * *P*<0.05, **P<0.01, using two-tailed Student t test

(A) mRNA expression levels of Collagen 1 and α-SMA were significantly decreased after indirubin (5 μM, 10 μM and 25 μM) treatment compared to TGF-β treatment group in HSC-T6 cells. The data are presented as mean ± SD. *P-*values: * *P*< 0.05, **P<0.01, using two-tailed Student t test. (B) Protein expression levels of Collagen 1 and α-SMA were significantly decreased after indirubin (5 μM, 10 μM and 25 μM) treatment compared to TGF-β treatment group in HSC-T6 cells

(A) Effects of Indirubin (12.5 mg/kg, 25 mg/kg) on the expression of p-Smad2/3, Smad2/3, p-p38, p38, p-ERK, ERK, p-JNK, JNK in the liver tissues were measured by WB analysis. (B) Effects of Indirubin (5 μM, 10 μM and 25 μM) on the expression of p-Smad2/3, Smad2/3, p-p38, p38, p-ERK, ERK, p-JNK, JNK in HSC-T6 cells were measured by WB analysis

## Discussion

Liver fibrosis is the excessive accumulation of ECM proteins that occurs in various chronic liver diseases (22, 23). Following chronic liver injury, activated HSCs trans-differentiate into proliferative myofibroblasts (7). Activated HSCs are the major source of ECM during the progression of liver fibrosis, and have been considered an attractive target for antifibrotic drug design (24, 25). However, the necessity of indirubin in this process has not been demonstrated. In this work, we confirmed the protective effect of indirubin on CCl_4_-triggered liver fibrosis and activation of HSC-T6 cells. We found that indirubin could significantly alleviate the degree of liver fibrosis and the trans-differentiation of HSC-T6 cells. Furthermore, our results suggested that indirubin reduced liver fibrosis and HSCs activation mainly through TGF-β-mediated signaling pathways *in vivo *and *in vitro*.

Indirubin, an active ingredient extracted from the leaves of Indigo Naturalis, has been used to treat CML, non-small cell lung carcinoma, osteosarcoma, and ovarian cancer (19, 20, 26, 27). Interestingly, a recent report indicated that indirubin alleviated bleomycin-induced pulmonary fibrosis (PF) in mice by inhibiting fibroblast to myofibroblast differentiation (16). However, whether indirubin could be used to treat liver fibrosis remains unclear. Firstly, we used the well-established rodent model of CCl_4_-induced liver fibrosis. Using this model, we found that indirubin therapy could attenuate liver injury and significantly down-regulate α-SMA and collagen 1 in the livers of mice, compared with controls. In particular, the therapeutic effect of indirubin on liver fibrosis was dose-dependent. Secondly, we used TGF-β-treated HSC-T6 cell-based models and found that indirubin could significantly inhibit HSCs trans-differentiation into myofibroblasts. Finally, we used the above different *in vivo *and *in vitro* models to elucidate the molecular mechanism of indirubin against liver fibrosis and HSC activation. We detected the changes in the TGF-β/Smad signaling pathway (p-Smad2/3) and TGF-β-mediated MAPK signaling pathway (p38, p-ERK, and p-JNK), and the results showed that indirubin could significantly inhibit the activities of p-Smad2/3, p-p38, p-ERK, and p-JNK *in vivo *and *in vitro*. These data suggested that indirubin might target the TGF-β-mediated signaling pathways to prevent liver fibrosis *in vivo *and *in vitro*.

In this study, we also found that while high concentration (10 μM or 25 μM) indirubin could markedly inhibit the viability and cell proliferation of HSC-T6 cells, low concentration (5 μM) indirubin was promoted. This suggested that the effect of indirubin on HSC-T6 cells was dose-dependent. In addition, how indirubin is involved in TGF-β signaling still needs further clarification.

## Conclusion

We indicated that indirubin could protect the mice from CCl_4_-induced liver fibrosis by regulating TGF-β-mediated signaling pathways. Taken together, our data show that indirubin could be a promising clinical therapeutic drug for the prevention and treatment of liver fibrosis.

## Authors’ Contributions

L W conceived, designed, and conducted the experiments. YZ Y and XY L conducted the experiments. YZ Y analyzed data and wrote the manuscript. L W administrated and supervised the project, and reviewed and edited the manuscript. All authors read and approved the final version of the manuscript.

## Data Availability

Not applicable.

## Conflicts of Interest

The authors declare that they have no conflicts of interest.
